# Comparative effectiveness of neoadjuvant treatment strategies and predictors of pathological complete response in locally advanced esophageal squamous cell carcinoma: a real-world retrospective cohort study

**DOI:** 10.3389/fonc.2026.1916194

**Published:** 2026-10-13

**Authors:** Xiaoli Yuan, Yuying Liang, Tianlin Han, Zhilei Guo, Yue Sun, Yifan Dong, Lu Zhang, Huahua Li, Jia Li, Xu Sun, Huaimin Liu

**Affiliations:** 1 The First Affiliated Hospital of Zhengzhou University, Zhengzhou, China; 2 The Second Clinical Medical College, Henan University of Chinese Medicine, Zhengzhou, China; 3 Department of Integrated Chinese and Western Medicine, Affiliated Cancer Hospital of Zhengzhou University and Henan Cancer Hospital, Zhengzhou, China; 4 Department of Medical Oncology, Affiliated People’s Hospital of Fujian University of Traditional Chinese Medicine, Fuzhou, China

**Keywords:** esophageal squamous cell carcinoma, logistic regression, neoadjuvant immunotherapy, pathological complete response, retrospective study

## Abstract

**Objective:**

To compare pathological complete response (pCR) across different neoadjuvant treatment strategies in patients with locally advanced esophageal squamous cell carcinoma (ESCC) and to identify clinicopathological factors associated with pCR.

**Methods:**

In this single-center retrospective cohort study, 247 consecutive patients with locally advanced ESCC who underwent neoadjuvant therapy followed by radical esophagectomy between January 2024 and June 2025 were included. Patients were categorized into four treatment groups: chemotherapy alone (CT, n = 50), chemoradiotherapy (CRT, n = 38), chemotherapy plus immunotherapy (CT+IO, n = 142), and chemoradiotherapy plus immunotherapy (CRT+IO, n = 17). Pathological complete response was defined as ypT0N0. Clinicopathological characteristics were compared using χ² or Fisher’s exact tests. Factors associated with pCR were evaluated using univariable and multivariable logistic regression analysis.

**Results:**

Overall, 63 patients (25.5%) achieved pCR. The pCR rate was lower in the CT group than in the CT+IO group (6.0% vs. 26.8%). After adjustment for potential confounders, chemotherapy alone remained associated with lower odds of pCR than CT+IO (OR = 0.174, 95% CI, 0.046-0.658, P = 0.010). No significant differences were observed between CT+IO and either CRT or CRT+IO. Receipt of more than two neoadjuvant treatment cycles was associated with higher odds of pCR (OR = 2.764, 95% CI, 1.372-5.565, P = 0.004). Compared with cT3 disease, cT2 disease was associated with higher odds of pCR (OR = 3.049, 95% CI, 1.356-6.857, P = 0.007).

**Conclusions:**

Neoadjuvant treatment regimen, the number of neoadjuvant treatment cycles, and pretreatment clinical T stage were associated with pathological complete response in locally advanced ESCC. Chemotherapy alone was associated with lower odds of pCR than CT+IO, whereas receipt of more than two treatment cycles was associated with higher odds of pCR. These findings provide real-world evidence regarding factors associated with pathological response.

## 1 Introduction

Esophageal cancer remains a major global health burden and is among the leading causes of cancer-related mortality worldwide (1). China bears a substantial proportion of the global esophageal cancer burden, with ESCC representing the predominant histological subtype (2, 3). Despite advances in surgical techniques and perioperative management, the prognosis of locally advanced ESCC remains unsatisfactory due to high rates of locoregional recurrence and distant metastasis. Most patients are diagnosed at potentially resectable locally advanced stages, generally including cT2–4a, any N, M0 disease, where surgery alone is associated with limited long-term survival and low R0 resection rates (4). A small number of patients with cT1N+M0 disease may also receive neoadjuvant therapy following multidisciplinary evaluation. Consequently, neoadjuvant therapy followed by radical esophagectomy has become an established treatment strategy for selected patients with locally advanced ESCC, as reflected in major clinical guidelines (5).Among these approaches, neoadjuvant chemoradiotherapy (nCRT) has been established as the historical standard based on landmark trials such as the CROSS and NEOCRTEC5010 studies, which demonstrated pathological complete response (pCR) rates of approximately 20%–43% and significant survival improvement compared with surgery alone (6, 7). However, nCRT may be associated with treatment-related toxicity and perioperative morbidity, which can limit its applicability in selected patients (8). In contrast, neoadjuvant chemotherapy (nCT) is better tolerated but yields relatively low pCR rates (typically 2%–9%) and limited long-term survival benefit (9). In recent years, immune checkpoint inhibitors (ICIs) targeting PD-1/PD-L1 have demonstrated remarkable efficacy in advanced ESCC, fundamentally transforming systemic treatment paradigms (10, 11). This success has rapidly extended into the perioperative setting, leading to growing interest in neoadjuvant immunotherapy strategies (12). Theoretically, neoadjuvant immunotherapy can activate broader and more durable tumor-specific T-cell responses in the presence of high tumor antigen load, potentially eradicating both primary lesions and micrometastases while inducing stronger immunological memory (13). Emerging clinical trials, including PALACE-1 (pembrolizumab combined with CROSS chemoradiotherapy, pCR rate 55.6%) (14), the CRIS study (neoadjuvant chemoradiotherapy followed by sequential tislelizumab, preliminary pCR rate 63.2%) (15), and a pooled analysis of 17 phase I/II trials (summary pCR rate approximately 48.7%) (16), have reported promising pCR rates ranging from 40% to over 60% for immunotherapy-containing regimens. Despite these encouraging results, several critical questions remain unresolved in the era of immunotherapy. First, the comparative effectiveness of different neoadjuvant strategies—including chemotherapy alone, nCRT, chemotherapy plus immunotherapy (nICT), and trimodality regimens combining chemoradiotherapy with immunotherapy—has not been fully clarified in real-world clinical practice. Second, reliable predictors of pathological response remain insufficiently defined, particularly regarding the roles of clinical T stage, nodal status, and treatment intensity. Third, the optimal number of neoadjuvant treatment cycles remains uncertain. Previous studies comparing different numbers of neoadjuvant treatment cycles have reported inconsistent findings regarding pathological response and treatment-related toxicity (17–19). Moreover, most existing evidence is derived from highly selected clinical trial populations, limiting generalizability to routine clinical practice. Real-world data evaluating heterogeneous patient populations, multiple immunotherapy agents, and varied treatment strategies remain scarce.

Therefore, this study aimed to retrospectively analyze data from patients with locally advanced ESCC who received neoadjuvant therapy at Henan Cancer Hospital, using logistic regression models to evaluate pCR rates across different neoadjuvant regimens and to identify factors associated with pCR, with the goal of providing real-world evidence and a clinical basis for individualized decision-making in neoadjuvant immunotherapy.

## 2 Materials and methods

### 2.1 Study design and patients

We conducted a single-center retrospective cohort study at Henan Cancer Hospital. Consecutive patients with histopathologically confirmed esophageal squamous cell carcinoma (ESCC) who underwent neoadjuvant therapy followed by radical esophagectomy between January 1, 2024, and June 30, 2025, were screened for eligibility.

### 2.2 Inclusion and exclusion criteria

Inclusion criteria:

(1) age ≥18 years with ESCC confirmed by histopathological examination of endoscopic biopsy specimens; (2) clinically diagnosed as locally advanced disease according to the cTNM staging system before neoadjuvant therapy, with complete baseline clinical and pathological data; (3) considered suitable candidates for neoadjuvant therapy and subsequently underwent radical esophagectomy after the completion of standardized neoadjuvant treatment.

Clinical staging was determined according to the eighth edition of the AJCC/UICC TNM staging system using endoscopic ultrasonography and contrast-enhanced chest and abdominal computed tomography, with positron emission tomography/computed tomography performed when clinically indicated. The four patients with cT1 disease had clinically positive regional lymph nodes (cT1N+M0) and received neoadjuvant therapy following multidisciplinary team evaluation.

Exclusion criteria:

(1) Previous anti-neoplastic treatment before the start of neoadjuvant therapy; (2) History of other primary malignant neoplasm. (3) Incomplete clinicopathological information.

### 2.3 Neoadjuvant treatment regimens

Treatment strategies were determined through multidisciplinary discussion according to tumor characteristics, disease stage, and the patient’s overall clinical condition. Treatment allocation was not randomized, and treatment selection may have been influenced by patient characteristics, treatment tolerance, physician judgment, and other clinical considerations. Therefore, comparisons between treatment groups were interpreted as observational associations rather than causal treatment effects. Patients received one of four neoadjuvant treatment regimens in routine clinical practice: chemotherapy alone (CT), chemoradiotherapy (CRT), chemotherapy plus immunotherapy (CT+IO), or chemoradiotherapy plus immunotherapy (CRT+IO).Immune checkpoint inhibitors included toripalimab, envafolimab, sintilimab, adebrelimab, pembrolizumab, tislelizumab, and camrelizumab, all administered according to the manufacturers’ recommended dosing schedules. All chemotherapy regimens consisted of paclitaxel combined with a platinum-based agent. Concurrent radiotherapy was delivered according to institutional treatment protocols. Patients received two to four cycles of neoadjuvant therapy and were categorized as receiving ≤2 or >2 cycles according to the number of chemotherapy cycles administered. Four to six weeks after completion of neoadjuvant therapy, all patients underwent comprehensive preoperative evaluation, followed by radical esophagectomy in the absence of surgical contraindications.

### 2.4 Surgical procedure and pathological evaluation

Radical esophagectomy was performed by experienced thoracic surgeons in accordance with standard oncologic principles, including radical lymph node dissection and tumor-free margin resection. The surgical approach was individualized based on tumor location, extent of disease, and the patient’s general condition. All resected specimens underwent standardized pathological examination by dedicated gastrointestinal pathologists. Pathological complete response (pCR) was defined as ypT0N0 on postoperative pathological evaluation.

### 2.5 Data collection and study endpoint

Baseline clinicopathological characteristics were extracted from the institutional electronic medical record system using a standardized case report form.Variables collected included age, sex, smoking history, alcohol consumption, body mass index (BMI), tumor location, pre-treatment clinical TNM stage (cTNM), neoadjuvant treatment regimen, number of treatment cycles, postoperative pathological stage, and pathological complete response. The primary endpoint of the study was pathological complete response (pCR) following neoadjuvant therapy. Associations between clinicopathological characteristics, neoadjuvant treatment strategies, and pCR were subsequently evaluated.

### 2.6 Statistical analysis

All statistical analysis were performed using SPSS version 27.0 (IBM Corp., Armonk, NY, USA). Categorical variables were presented as counts and percentages, and comparisons between groups were made using the Pearson chi-square test or Fisher’s exact test, as appropriate. Univariate logistic regression was used to screen variables associated with pCR, and variables with P < 0.05 in the univariate analysis were entered into a multivariate logistic regression model to identify independent influencing factors for pCR. The Hosmer-Lemeshow goodness-of-fit test was used to assess model calibration. All tests were two-sided, and P < 0.05 was considered statistically significant.

## 3 Results

### 3.1 Patient selection

A total of 274 consecutive patients with histopathologically confirmed esophageal squamous cell carcinoma (ESCC) who received neoadjuvant therapy followed by radical esophagectomy at Henan Cancer Hospital were screened for eligibility during the study period. Of these, 27 patients were excluded for the following reasons: previous antitumor therapy (n=15), history of other primary malignant tumors (n=11), and incomplete clinicopathological data (n=1). The patient selection process is summarized in **Figure 1**.

**Figure 1:**
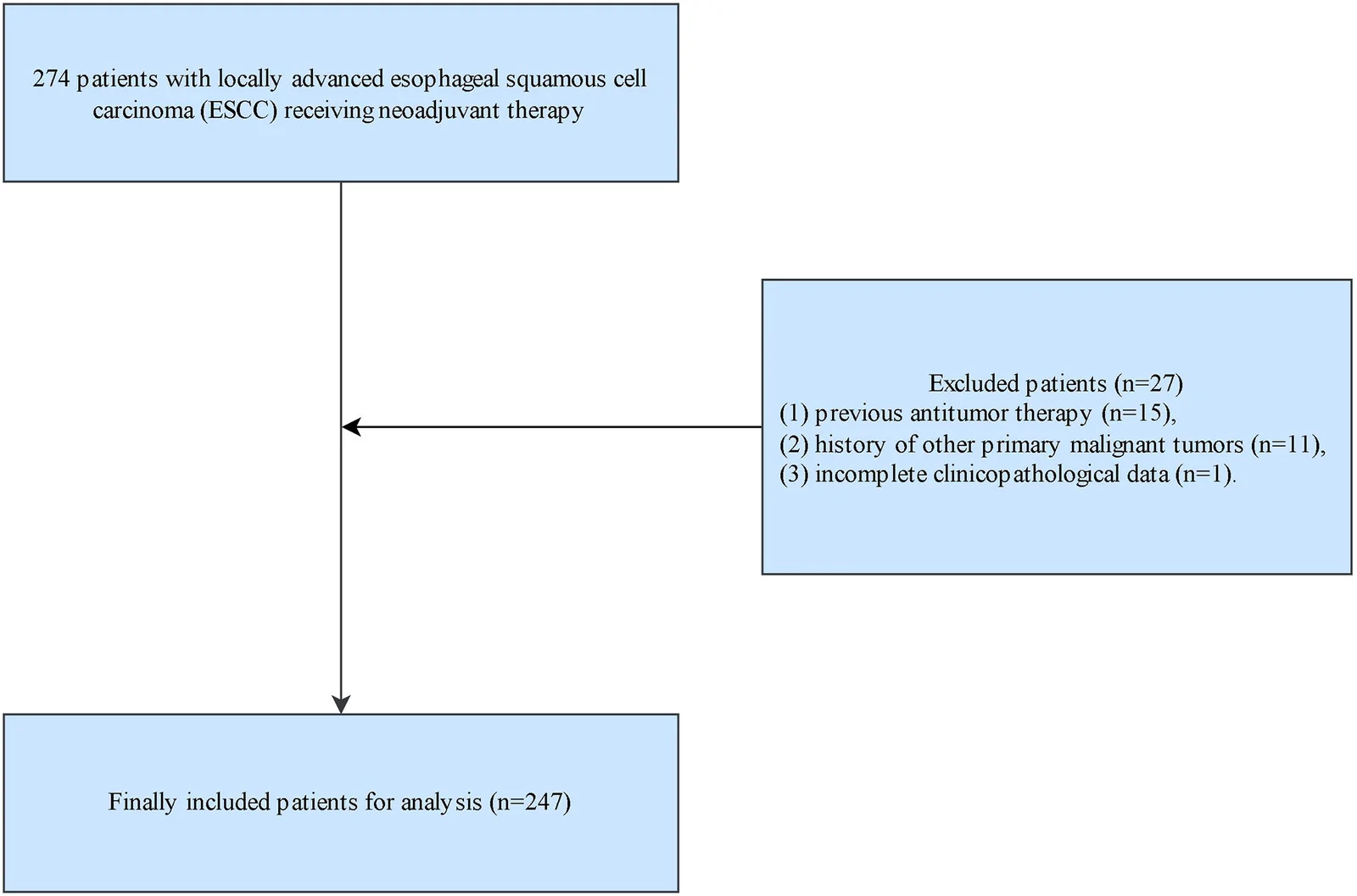
Flowchart of patient enrollment and screening in this study.

### 3.2 Baseline characteristics of the study population

The study cohort consisted predominantly of male patients with clinically staged cT3 disease, and approximately one-quarter of the patients achieved pathological complete response (pCR) following neoadjuvant therapy. Among the 247 eligible patients, 178 (72.1%) were male and 69 (27.9%) were female. A total of 132 patients (53.4%) were older than 65 years, while 137 (55.5%) had a body mass index (BMI) between 18.5 and 24.0 kg/m². Primary tumors were most frequently located in the middle thoracic esophagus (125 patients, 50.6%), followed by the lower thoracic (96, 38.9%) and upper thoracic esophagus (26, 10.5%).With respect to pretreatment clinical staging, cT3 disease was the predominant tumor stage (180 patients, 72.9%), whereas cN1 disease represented the most common nodal stage (144 patients, 58.3%).Regarding neoadjuvant treatment, chemotherapy plus immunotherapy (CT+IO) was the most frequently administered regimen, accounting for 142 patients (57.5%), followed by chemotherapy alone (50, 20.2%), chemoradiotherapy (38, 15.4%), and chemoradiotherapy plus immunotherapy (17, 6.9%). Most patients (162, 65.6%) received two or fewer cycles of neoadjuvant therapy. Following neoadjuvant treatment and radical esophagectomy, 63 patients (25.5%) achieved pathological complete response (pCR), whereas 184 patients (74.5%) had residual pathological disease. The baseline demographic and clinicopathological characteristics of the study population are summarized in **Table 1**, and the distribution of patients according to pCR status is illustrated in **Figure 2**.

**Table 1:** Clinical and pathological characteristics of the study population (n = 247).

Characteristic	Number of patients(N=247)	Percentage (%)
Age		
≤65 years	115	46.6%
>65 years	132	53.4%
Sex		
Male	178	72.1%
Female	69	27.9%
BMI		
<18.5kg/m²	21	8.5%
18.5~24.0kg/m²	137	55.5%
>24.0kg/m²	89	36.0%
Smoking history		
No	136	55.1%
Yes	111	44.9%
Alcohol consumption		
No	144	58.3%
Yes	103	41.7%
Tumor location		
Upper thoracic	26	10.5%
Middle thoracic	125	50.6%
Lower thoracic	96	38.9%
Neoadjuvant regimen		
CT	50	20.2%
CRT	38	15.4%
CT+IO	142	57.5%
CRT+IO	17	6.9%
Treatment cycles		
≤2	162	65.6%
>2	85	34.4%
Clinical T stage		
T1	4	1.6%
T2	46	18.6%
T3	180	72.9%
T4	17	6.9%
Clinical N stage		
N0	69	27.9%
N1	144	58.3%
N2	34	13.8%
ypT stage		
T0	72	29.2%
T1	57	23.1%
T2	44	17.8%
T3	64	25.9%
T4	10	4.0%
ypN stage		
N0	166	67.2%
N1	54	21.9%
N2	22	8.9%
N3	5	2.0%
pCR		
patients with pCR	63	25.5%
patients without pCR	184	74.5%

CT, Chemotherapy; CRT, Radiotherapy plus chemotherapy; CT+IO, Chemotherapy plus immunotherapy; CRT+IO, Radiotherapy plus chemotherapy and immunotherapy (the same below).

**Figure 2:**
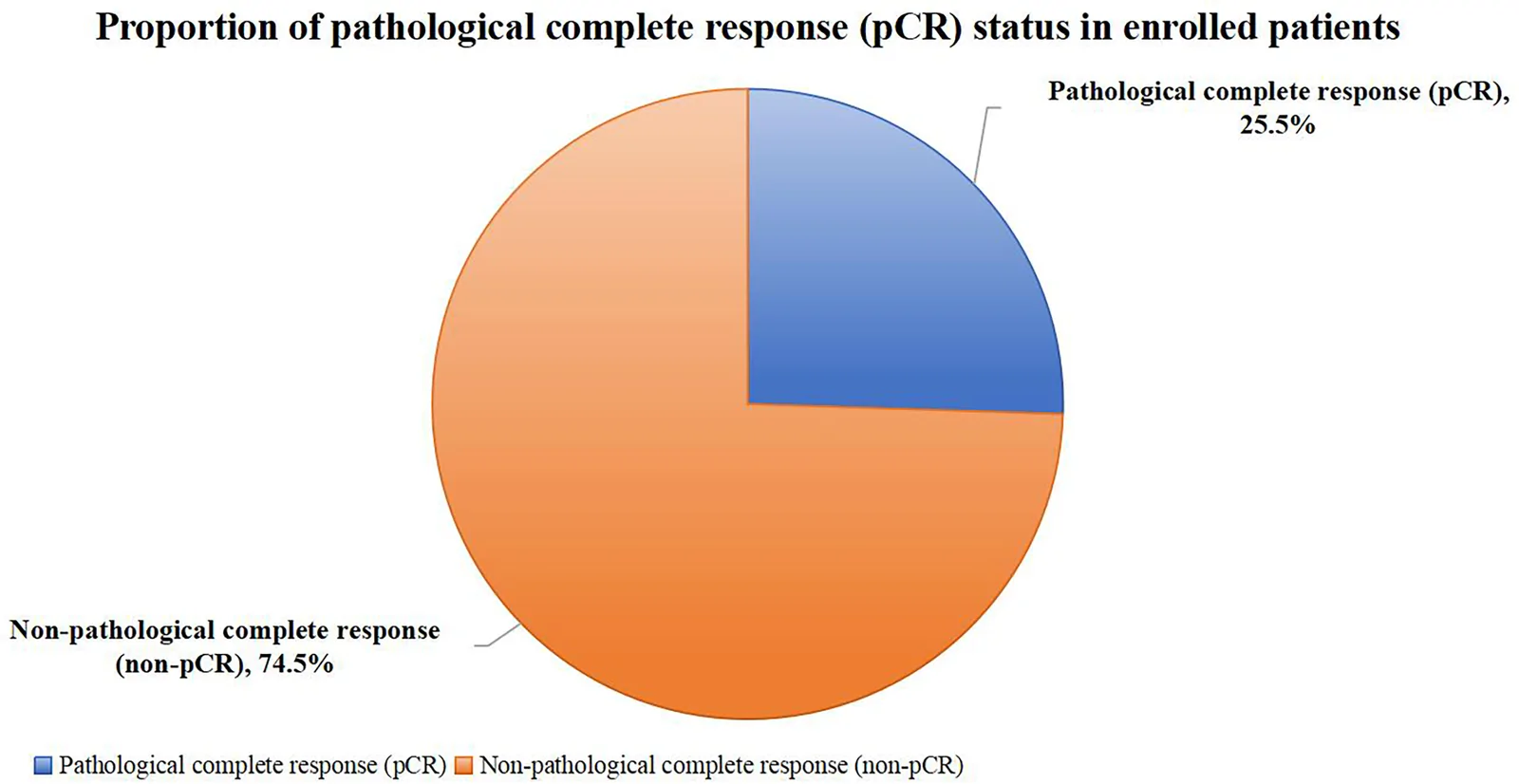
Pie chart showing the composition of pCR and non-pCR cases among enrolled patients.

### 3.3 Comparison of baseline characteristics across neoadjuvant treatment groups

The 247 patients were classified according to the neoadjuvant regimen received into four treatment groups: chemotherapy alone (CT, n = 50), chemoradiotherapy (CRT, n = 38), chemotherapy plus immunotherapy (CT+IO, n = 142), and chemoradiotherapy plus immunotherapy (CRT+IO, n = 17). No statistically significant differences were detected among the four groups with respect to sex, age, smoking history, alcohol consumption, body mass index (BMI), primary tumor location, pretreatment clinical T stage (cT), or clinical N stage (cN) (all P > 0.05). In contrast, the distribution of neoadjuvant treatment cycles differed significantly among the four treatment groups (P < 0.001). Specifically, patients treated with chemotherapy alone were more likely to receive two or fewer treatment cycles, whereas a greater proportion of patients in the chemoradiotherapy group received more than two cycles. The CT+IO and CRT+IO groups showed intermediate distributions. Given the significant difference in treatment cycle number across the four treatment groups, this variable was included in the subsequent multivariable logistic regression analysis. Detailed comparisons of baseline demographic and clinicopathological characteristics among the four treatment groups are presented in **Table 2**.

**Table 2:** Comparison of baseline patient characteristics between different neoadjuvant therapy groups, n (%).

Characteristic	Sub-category	Neoadjuvant regimen				*χ²*	*P*
		CT (n=50)	CRT (n=38)	CT+IO (n=142)	CRT+IO (n=17)		
Sex	Male	40 (80.0)	28 (73.7)	98 (69.0)	12 (70.6)	-	0.516
	Female	10 (20.0)	10 (26.3)	44 (31.0)	5 (29.4)		
Age	≤65 years	23 (46.0)	19 (50.0)	62 (43.7)	11 (64.7)	2.916	0.405
	>65 years	27 (54.0)	19 (50.0)	80 (56.3)	6 (35.3)		
Smoking history	No	25 (50.0)	24 (63.2)	78 (54.9)	9 (52.9)	1.556	0.669
	Yes	25 (50.0)	14 (36.8)	64 (45.1)	8 (47.1)		
Alcohol consumption	No	26 (52.0)	20 (52.6)	88 (62.0)	10 (58.8)	2.108	0.550
	Yes	24 (48.0)	18 (47.4)	54 (38.0)	7 (41.2)		
BMI	<18.5kg/m²	4 (8.0)	5 (13.2)	11 (7.7)	1 (5.9)	-	0.573
	18.5~24.0kg/m²	24 (48.0)	24 (63.2)	79 (55.6)	10 (58.8)		
	>24.0kg/m²	22 (44.0)	9 (23.7)	52 (36.6)	6 (35.3)		
Tumor location	Upper thoracic	5 (10.0)	7 (18.4)	14 (9.9)	0 (0.0)	-	0.181
	Middle thoracic	20 (40.0)	17 (44.7)	76 (53.5)	12 (70.6)		
	Lower thoracic	25 (50.0)	14 (36.8)	52 (36.6)	5 (29.4)		
Treatment cycles	≤2	44 (88.0)	12 (31.6)	97 (68.3)	9 (52.9)	32.271	<0.001
	>2	6 (12.0)	26 (68.4)	45 (31.7)	8 (47.1)		
Clinical T stage	1	1 (2.0)	0 (0.0)	3 (2.1)	0 (0.0)	-	0.827
	2	7 (14.0)	6 (15.8)	30 (21.1)	3 (17.6)		
	3	36 (72.0)	30 (78.9)	100 (70.4)	14 (82.4)		
	4	6 (12.0)	2 (5.3)	9 (6.3)	0 (0.0)		
Clinical N stage	0	17 (34.0)	9 (23.7)	41 (28.9)	2 (11.8)	-	0.241
	1	27 (54.0)	24 (63.2)	84 (59.2)	9 (52.9)		
	2	6 (12.0)	5 (13.2)	17 (12.0)	6 (35.3)		

### 3.4 Logistic regression analysis of factors associated with pCR

To identify clinicopathological factors independently associated with pathological complete response (pCR), univariable logistic regression analysis were initially performed for all prespecified baseline variables. Variables demonstrating statistical significance in the univariable analysis were subsequently entered into a multivariable logistic regression model to determine independent predictors of pCR. The variable coding scheme used for model construction is summarized in **Table 3**.

**Table 3:** Variable assignment for logistic regression analysis.

Variable	Assignment method
Sex	Male = 0, Female = 1
Age	≤65 years = 0, >65 years = 1
Smoking history	No smoking history = 0, Smoking history = 1
Alcohol consumption	No drinking history = 0, Drinking history = 1
BMI	<18.5 = 2, 18.5-24 = 1, >24 = 3
Tumor location	Upper thoracic = 2, Middle thoracic = 1, Lower thoracic = 3
Neoadjuvant therapy	CT+IO = 1, CT = 2, CRT = 3, CRT+IO = 4
Treatment cycles	≤2 = 0, >2 = 1
Clinical T stage	T3 = 1, T1 = 3, T2 = 2, T4 = 4
Clinical N stage	N0 = 1, N1 = 3, N2 = 2
pCR	ypT0N0 = 1, non-pCR= 0

Variables with more than two categories were entered into the logistic regression model as categorical variables using indicator coding rather than as continuous ordinal variables.

#### 3.4.1 Univariable logistic regression analysis

Univariable logistic regression analysis identified neoadjuvant treatment regimen, number of treatment cycles, and pretreatment clinical T stage as variables potentially associated with pathological complete response, whereas no significant associations were observed for the remaining baseline clinicopathological characteristics. Using chemotherapy plus immunotherapy (CT+IO) as the reference regimen, patients who received chemotherapy alone (CT) had significantly lower odds of achieving pCR (OR = 0.175, 95% CI, 0.051-0.595; P = 0.005). The number of neoadjuvant treatment cycles was also associated with pCR in the univariable analysis. Compared with patients who received ≤2 treatment cycles, those who received >2 cycles were more likely to achieve pCR (OR = 3.350, 95% CI, 1.850-6.066; P < 0.001). Among pretreatment tumor stages, cT2 disease was associated with significantly higher odds of achieving pCR than cT3 disease (OR = 2.485, 95% CI, 1.242-4.972; P = 0.010). In addition, cT4 disease was associated with higher odds of achieving pCR in the univariable analysis (OR = 3.746, 95% CI, 1.205-11.648; P = 0.023), but with a relatively wide confidence interval. No statistically significant associations were identified for age, sex, body mass index, smoking history, alcohol consumption, primary tumor location, chemoradiotherapy (CRT), chemoradiotherapy plus immunotherapy (CRT+IO), cT1 stage, or pretreatment clinical N stage (all P > 0.05). Detailed results of the univariable logistic regression analysis are presented in **Table 4**.

**Table 4:** Univariate logistic regression analysis of factors associated with pathological complete response (pCR).

Characteristic	*OR*	95%CI	*P*
Age	(reference: ≤65 years)		
>65 years	0.564	0.317-1.006	0.052
Sex	(reference: male)		
Female	1.417	0.763-2.630	0.270
BMI	(reference: 18.5-24.0kg/m²)		
<18.5kg/m²	0.947	0.323-2.778	0.921
>24.0kg/m²	1.119	0.609-2.054	0.718
Smoking history	(reference: no)		
Yes	0.893	0.501-1.590	0.700
Alcohol consumption	(reference: no)		
Yes	0.748	0.414-1.348	0.334
Tumor location	(reference: middle)		
Upper	0.771	0.286-2.081	0.608
Lower	0.764	0.413-1.415	0.393
Neoadjuvant regimen	(reference: CT+IO)		
CT	0.175	0.051-0.595	0.005
CRT	1.990	0.946-4.186	0.070
CRT+IO	1.493	0.516-4.317	0.460
Treatment cycles	(reference: ≤2)		
>2	3.350	1.850-6.066	<0.001
Clinical T stage	(reference: cT3)		
T1	1.288	0.130-12.745	0.828
T2	2.485	1.242-4.972	0.010
T4	2.705	0.965-7.588	0.059
Clinical N stage	(reference: cN1)		
N0	0.758	0.390-1.476	0.416
N2	0.538	0.208-1.396	0.203

#### 3.4.2 Multivariable logistic regression analysis

Variables that were statistically significant in univariate analysis or considered clinically relevant were included in the multivariate logistic regression model. Model fit evaluation showed that the overall model was statistically significant (Omnibus test: χ² = 46.638, df = 17, P < 0.001); the Hosmer–Lemeshow test indicated good model fit (χ² = 7.971, df = 8, P = 0.436). In the multivariable analysis, neoadjuvant treatment regimen, treatment cycle number, and pretreatment clinical T stage were associated with pCR. Relative to the CT+IO group, patients treated with chemotherapy alone remained significantly less likely to achieve pCR (OR = 0.174, 95% CI, 0.046-0.658; P = 0.010). By contrast, no statistically significant differences were observed between CT+IO and either CRT or CRT+IO (both P > 0.05). Treatment cycle number remained associated with pCR in the multivariable analysis. Patients who received more than two neoadjuvant treatment cycles had significantly higher odds of achieving pCR than those receiving two or fewer cycles (OR = 2.764, 95% CI, 1.372-5.565; P = 0.004). Pretreatment clinical T stage was also independently associated with pathological response. Compared with cT3 disease, patients with cT2 disease had significantly higher odds of achieving pCR (OR = 3.049, 95% CI, 1.356-6.857; P = 0.007). Similarly, cT4 disease was associated with higher odds of pCR (OR = 3.797, 95% CI, 1.103-13.070; P = 0.034), with a relatively wide confidence interval, indicating an imprecise estimate; therefore, it is not presented as a definitive finding. The complete multivariable logistic regression results are summarized in **Table 5**.

**Table 5:** Multivariable logistic regression analysis of factors associated with pathological complete response (pCR).

Characteristic	*OR*	95%*CI*	*P*
Neoadjuvant regimen	(reference: CT+IO)		
CT	0.174	0.046-0.658	0.010
CRT	1.868	0.775-4.505	0.164
CRT+IO	1.438	0.431-4.798	0.554
Treatment cycles	(reference: ≤2)		
>2	2.764	1.372-5.565	0.004
Clinical T stage	(reference: cT3)		
T1	1.264	0.101-15.835	0.856
T2	3.049	1.356-6.857	0.007
T4	3.797	1.103-13.070	0.034

### 3.5 Summary of findings

Overall, 247 patients with locally advanced ESCC who underwent neoadjuvant therapy followed by radical esophagectomy were included in the final analysis, and 63 patients (25.5%) achieved pathological complete response (pCR). Baseline demographic and clinicopathological characteristics were generally comparable across the four neoadjuvant treatment groups, with the exception of the number of neoadjuvant treatment cycles, which differed significantly among groups and was therefore accounted for in the multivariable analysis. In the multivariable logistic regression model, neoadjuvant treatment regimen, number of treatment cycles, and pretreatment clinical T stage were associated with pCR after adjustment for the covariates included in the model. Compared with CT+IO, chemotherapy alone was associated with lower odds of achieving pCR. Receipt of more than two neoadjuvant treatment cycles was associated with higher odds of pCR than receipt of two or fewer cycles. Compared with cT3 disease, cT2 disease was associated with higher odds of pCR. The subgroup analysis showed numerically higher pCR proportions in the cT2, >2 treatment cycles, and chemoradiotherapy-containing groups, whereas the chemotherapy-alone group had the lowest pCR rate.

## 4 Discussion

Esophageal cancer remains one of the leading causes of cancer-related mortality worldwide, and China bears a substantial proportion of the global disease burden, with ESCC representing the predominant histological subtype (1–3).For locally advanced ESCC, neoadjuvant chemoradiotherapy followed by radical esophagectomy remains the standard treatment strategy (10). In recent years, the expanding use of immunotherapy in advanced ESCC has prompted exploration of immunotherapy combined with chemotherapy in the neoadjuvant setting. Theoretically, neoadjuvant immunotherapy can activate more durable systemic antitumor immunity in the presence of rich tumor antigens, potentially further improving pathological response depth (13). However, real-world evidence regarding the comparative efficacy of different neoadjuvant strategies—including chemotherapy alone, concurrent chemoradiotherapy, chemotherapy combined with immunotherapy, and chemoradiotherapy combined with immunotherapy—and the identification of optimal candidates for immunotherapy-based regimens, remains insufficient. Against this background, the present retrospective cohort study evaluated the association between neoadjuvant treatment strategy and pCR in patients with locally advanced ESCC treated with radical esophagectomy following neoadjuvant therapy. Neoadjuvant treatment regimen, treatment cycle number, and pretreatment clinical T stage were associated with pCR in the multivariable analysis. These findings provide the framework for the following discussion.

### 4.1 Neoadjuvant treatment strategy and pathological response

Neoadjuvant treatment regimen was associated with pathological response in the multivariable analysis. Compared with CT+IO, chemotherapy alone was associated with significantly lower odds of achieving pCR after adjustment for treatment cycle number and clinical T stage. These findings are consistent with the growing body of evidence supporting the role of immunotherapy in the multidisciplinary management of ESCC. Recent systematic reviews and meta- analysis have reported higher pathological response rates with neoadjuvant chemoimmunotherapy than with chemotherapy alone in patients with resectable ESCC (20, 21), which is consistent with the direction of the association observed in the present study. The enhanced pathological response may reflect complementary mechanisms between cytotoxic chemotherapy and immune checkpoint inhibition, whereby chemotherapy promotes tumor antigen release while immunotherapy restores antitumor immune activity. In the present cohort, numerically higher pCR rates were also observed in patients receiving chemoradiotherapy-containing regimens, particularly CRT alone. However, neither CRT nor CRT+IO remained significantly different from CT+IO after multivariable adjustment. This discrepancy between crude response rates and adjusted estimates may reflect baseline clinical differences, limited subgroup sample sizes, and reduced statistical power after covariate adjustment (22). Because treatment allocation was non-randomized and determined by multidisciplinary clinical judgment, these findings represent observational associations rather than causal evidence of treatment superiority.

### 4.2 Treatment cycle number and pathological response

Another finding of the present study was that treatment cycle number remained associated with pCR after adjustment for treatment regimen and clinical T stage. Patients who received more than two cycles of neoadjuvant therapy were significantly more likely to achieve pCR than those who received two or fewer cycles. Although the magnitude of the association was attenuated after multivariable adjustment compared with the univariable analysis, the association remained statistically significant after adjustment for treatment regimen and other measured covariates. This observation is clinically plausible. Additional treatment cycles may prolong exposure to systemic therapy, thereby increasing cumulative tumor cell kill and allowing a more sustained antitumor immune response in patients receiving immunotherapy-containing regimens. Nevertheless, because the number of neoadjuvant treatment cycles in routine clinical practice is influenced by multiple factors—including treatment tolerance, clinical response, physician judgment, and patient preference—the association observed in this retrospective study should not be interpreted as evidence of a direct causal effect. Our findings are broadly consistent with a previous retrospective study reporting an association between receipt of three neoadjuvant chemoimmunotherapy cycles and improved pathological response compared with two cycles (23). However, the optimal number of neoadjuvant treatment cycles for ESCC remains uncertain, and existing comparative studies have not established a standard treatment cycle number (17–19). Consequently, the number of neoadjuvant treatment cycles should continue to be individualized according to treatment response, tolerability, and surgical feasibility.

### 4.3 Pretreatment clinical T stage and pathological response

Pretreatment clinical T stage was associated with pathological response in the present study. Compared with cT3 disease, cT2 disease was associated with higher odds of achieving pCR in the multivariable analysis. Taken together, these findings suggest that pretreatment clinical T stage may contribute to risk stratification and individualized treatment planning in patients with locally advanced ESCC. However, its predictive value should be considered alongside other clinicopathological characteristics rather than used in isolation when selecting neoadjuvant treatment strategies.

### 4.4 Other clinicopathological variables

In the present study, age, sex, BMI, smoking history, alcohol consumption, tumor location, and pretreatment clinical N stage were not significantly associated with pCR in the univariable analysis. Previous studies have investigated clinicopathological, inflammatory, and immune-related factors associated with pathological response after neoadjuvant therapy for ESCC; however, the reported predictors have not been consistent across different cohorts and treatment regimens (24, 25). Such discrepancies may reflect differences in patient selection, treatment strategies, staging methods, biomarker definitions, sample size, and statistical modeling. Of note, age demonstrated a borderline association with pCR in the univariable analysis but did not satisfy the predefined criterion for inclusion in the multivariable model. Although no independent association was identified in the present study, the potential influence of age on treatment response cannot be excluded and warrants further investigation in larger prospective datasets. Overall, the present findings suggest that conventional baseline demographic characteristics alone may provide limited information for predicting pathological response. Future predictive models may benefit from integrating clinicopathological variables with molecular biomarkers and treatment-related characteristics to improve individualized risk stratification.

### 4.5 Strengths of the study

Several strengths of the present study should be acknowledged. First, this study directly compared four contemporary neoadjuvant treatment strategies within the same real-world cohort, allowing evaluation of their relative associations with pathological response under routine clinical practice. Such comparative data remain limited in patients with locally advanced ESCC. Second, unlike analysis relying solely on crude subgroup comparisons, the present study incorporated multivariable logistic regression to adjust for important baseline differences, thereby improving the robustness of the estimated associations between clinicopathological variables and pCR. Third, pathological complete response was selected as the primary endpoint. Because pCR is an objective pathological outcome assessed after radical resection and has consistently been associated with favorable long-term survival in previous studies (26–29), it represents a clinically relevant short-term endpoint in neoadjuvant studies. Together, these strengths enhance the clinical relevance of the present findings and provide complementary real-world evidence regarding factors associated with pathological response in patients with locally advanced ESCC.

### 4.6 Clinical implications

Despite these limitations, the present study provides several clinically relevant observations. The findings suggest that neoadjuvant treatment regimen, number of neoadjuvant treatment cycles, and pretreatment clinical T stage may assist clinicians in estimating the probability of achieving pathological complete response before surgery. Importantly, these variables should not be regarded as independent treatment-selection criteria. Instead, they should be interpreted within a comprehensive multidisciplinary decision-making framework that also considers patient performance status, treatment tolerance, surgical feasibility, comorbidities, and patient preferences. From a translational perspective, the present results also support continued investigation of predictive models integrating conventional clinicopathological variables with molecular biomarkers to facilitate more precise selection of individualized neoadjuvant treatment strategies.

### 4.7 Limitations

This study has several limitations. First, its retrospective, single-center, and non-randomized design may have introduced selection bias and confounding by indication, which may limit the generalizability of the findings. Treatment allocation was based on multidisciplinary clinical judgment and may have been influenced by patient characteristics, treatment tolerance, physician judgment, and other clinical considerations. Although multivariable adjustment was performed, residual confounding from unmeasured clinical factors cannot be excluded. Second, some subgroups were small, particularly cT1 (n=4), cT4 (n=17), and chemoradiotherapy plus immunotherapy (n=17), resulting in imprecise effect estimates and limited statistical power for subgroup comparisons. Third, the use of seven different immune checkpoint inhibitors introduced treatment heterogeneity. Fourth, potentially relevant biomarkers, including PD-L1 expression and tumor immune microenvironment characteristics, were not routinely available, limiting assessment of biological heterogeneity in treatment response (25, 30). Fifth, the absence of long-term follow-up prevented evaluation of the relationship between pCR and oncological outcomes, including disease-free survival and overall survival. Finally, no formal correction for multiple comparisons was performed; therefore, the possibility of false-positive findings cannot be excluded. Taken together, these limitations indicate that the observed associations should be interpreted within the context of the study design and require validation in larger prospective multicenter cohorts.

## 5 Conclusion

In conclusion, this retrospective cohort study identified neoadjuvant treatment regimen, number of neoadjuvant treatment cycles, and pretreatment clinical T stage as factors associated with pathological complete response in patients with locally advanced ESCC undergoing neoadjuvant therapy followed by radical esophagectomy. Compared with chemotherapy plus immunotherapy, chemotherapy alone was associated with significantly lower odds of achieving pCR, whereas receipt of more than two treatment cycles and cT2 disease were associated with higher odds of pCR than their respective reference categories. Given the non-randomized treatment allocation and the observational nature of the study, these findings should not be interpreted as causal treatment effects. Larger prospective multicenter studies with standardized treatment protocols, integrated biomarker assessment, and long-term oncologic follow-up are needed to validate these observations.

## Data Availability

The original contributions presented in the study are included in the article/supplementary material. Further inquiries can be directed to the corresponding authors.
